# Acute and second-meal effects of almond form in impaired glucose tolerant adults: a randomized crossover trial

**DOI:** 10.1186/1743-7075-8-6

**Published:** 2011-01-28

**Authors:** Alisa M Mori, Robert V Considine, Richard D Mattes

**Affiliations:** 1 Department of Foods and Nutrition, Purdue University, West Lafayette, IN, USA; 2 Divison of Endocrinology, Department of Medicine, Indiana University, Indianapolis, IN, USA

## Abstract

**Background:**

Nut consumption may reduce the risk of developing type 2 diabetes. The aim of the current study was to measure the acute and second-meal effects of morning almond consumption and determine the contribution of different nut fractions.

**Methods:**

Fourteen impaired glucose tolerant (IGT) adults participated in a randomized, 5-arm, crossover design study where whole almonds (WA), almond butter (AB), defatted almond flour (AF), almond oil (AO) or no almonds (vehicle - V) were incorporated into a 75 g available carbohydrate-matched breakfast meal. Postprandial concentrations of blood glucose, insulin, non-esterified free fatty acids (NEFA), glucagon-like peptide-1 (GLP-1) and appetitive sensations were assessed after treatment breakfasts and a standard lunch.

**Results:**

WA significantly attenuated second-meal and daylong blood glucose incremental area under the curve (AUCI) and provided the greatest daylong feeling of fullness. AB and AO decreased blood glucose AUCI in the morning period and daylong blood glucose AUCI was attenuated with AO. WA and AO elicited a greater second-meal insulin response, particularly in the early postprandial phase, and concurrently suppressed the second-meal NEFA response. GLP-1 concentrations did not vary significantly between treatments.

**Conclusions:**

Inclusion of almonds in the breakfast meal decreased blood glucose concentrations and increased satiety both acutely and after a second-meal in adults with IGT. The lipid component of almonds is likely responsible for the immediate post-ingestive response, although it cannot explain the differential second-meal response to AB versus WA and AO.

## Background

The 2025 worldwide projection of IGT is 418 million (8.1% of the adult population) [1]. Lifestyle modification, including nutrition is the cornerstone of its management. Macro- and micronutrients, fiber content, and other components of the diet modulate meal-induced insulin secretion through changes in gastrointestinal transit time and nutrient absorption rates. Additionally, the content of one meal has the potential to affect insulin sensitivity at a second meal by altering circulating NEFA concentrations and daylong insulin demands [2].

Almonds are a low-glycemic index (GI) food, with high fiber, unsaturated fat and low carbohydrate content. There is an inverse relationship between nut consumption and risk of developing type 2 diabetes [3]. In addition, almond consumption increases satiety, reduces cardiovascular disease risk, decreases postprandial glycemia and moderates oxidative damage [4]. The component(s) of almonds responsible for these effects have not been determined. Almonds contain phytates and phenolics, that confer antioxidant, anti-inflammatory and lipid-lowering properties and inhibit trypsin and amylase activity [5]. A decreased rate of nutrient digestion may explain reported increases in satiety and blunted blood glucose response with almond consumption. Stimulation of the incretin and ileal-brake hormone, GLP-1, may also contribute. Consequently, almond consumption may be an effective dietary management tool in insulin resistant individuals who would benefit from replacement of saturated fat with unsaturated fat [6].

Inclusion of 60 g of almonds in meals of healthy individuals decreases glycemia, insulinemia, and postprandial oxidative damage as measured by increased protein thiol concentration [7]. Adding almonds (30-90 g) to a high-GI meal results in a dose-responsive decrease in 2-hour postprandial blood glucose AUCI [8]. However, consumption of almond oil with defatted almond flour, to mimic a bioaccessible almond form, significantly decreased 3-hour blood glucose AUCI with no difference in insulin response compared to when small almond particles were consumed [9]. Similarly, increased and sustained concentrations of cholecystokinin (CCK) and augmented hunger were reported with bioavailable almond oil compared to whole almonds [10]. This suggests the bioavailability of the lipid fraction may be responsible for decreased postprandial glycemia.

The present study evaluated the effects of whole almonds, almond oil, defatted almond flour, and almond butter on acute and second-meal postprandial blood glucose, insulin, NEFA, and GLP-1 concentrations, as well as satiety sensations, in IGT adults.

## Methods

Eligibility criteria included: age 18-60 years; not taking medications known to affect glycemia, sleep, or appetite; weight stable (3 month fluctuation of <5 kg); regular breakfast consumer (≥100 kcal ingested within 2 hours of waking on ≥4 d/wk) o blood donation in the previous 3 months; no nut or relevant food allergy; at least one of the following risk factors: A) self-reported family history of type 2 diabetes; B) blood pressure ≥130/85 mmHg; C) fasting blood glucose >5.6 mmol/l; or D) waist circumference (men ≥102 cm; women ≥88 cm); and a 2-hour blood glucose value of 7.8 and <11.1 mmol/l (i.e., IGT) [11]. Height, weight, and body composition were measured using a wall-mounted stadiometer, a clinical scale, and bioelectrical impedence, respectively. A 2-hour, 75-gram oral glucose tolerance test (OGTT) was conducted at a second visit with participants in an 8-10 hour fasted state. The research was approved by the University Institutional Review Board.

One hundred-seventy individuals completed the first screening visit, of which 132 were eligible for and completed the second screening visit. Fourteen participants met all screening criteria and completed the full study protocol. Calculation of power indicated that 13 individuals were necessary to detect a change in blood glucose of 0.35 mmol/l (α = 0.05; Power = 0.80, SD = 0.3)[12]. Participant characteristics are shown in Table 1.

**Table 1:** Participant characteristics¹

Age (y)	39.3 ± 10.9
Weight (kg)	92.6 ± 19.3

BMI (kg/m²)	33.0 ± 6.9

Body fat (%)	35.8 ± 14.0

Waist circumference (cm)	105.3 ± 16.3

Systolic blood pressure (mmHg)	130.1 ± 11.1

Diastolic blood pressure (mmHg)	82.0 ± 10.0

Fasting blood glucose (mmol/l)	5.5 ± 0.5

Fasting serum insulin (pmol/l)	88.8 ± 46.2

Total cholesterol (mmol/l)	5.42 ± 1.14

HDL-C (mmol/l)	1.18 ± 0.45

LDL-C (mmol/l)	3.16 ± 1.07

Cholesterol:HDL-C ratio	5.1 ± 1.8

Triglycerides (mmol/l)	2.80 ± 1.37

Blood glucose after 2-hour OGTT (mmol/l)	8.3 ± 0.3

QUICKI²	0. 330 ± 0.039

^1^N = 14 (8 M, 6 F); Mean ± SD

^2^Quantitative insulin sensitivity check index = 1/[log (fasting insulin, μU/mL) + log (fasting glucose, mg/dL)] [14]

The study utilized a randomized, 5-arm, crossover, single-blinded design. Overnight fasted (8-10 hours) participants reported to the laboratory on 5 occasions separated by at least one week. Menstruating female participants completed test days within the follicular phase of their menstrual cycle. Individuals were requested to maintain their normal exercise, eating, and sleeping patterns and, if taking vitamins or medications, to take them at the same time every day before reporting for testing. Participants were also requested to consume the same meal each evening before reporting to the laboratory at their customary breakfast time.

Upon arrival to the laboratory, participants were weighed and body composition was determined. An indwelling catheter was placed and a baseline blood sample collected. Appetite ratings were scored on a 100 mm visual analogue scale (VAS) anchored with descriptors of "not at all" and "extremely." Next, the participant consumed a test breakfast within 10 minutes that consisted of orange juice and farina [prepared Cream of Wheat^®^, B&G Foods, Inc.] containing: nothing V (vehicle), whole almonds (WA), almond butter (AB), defatted almond flour (AF) or almond oil (AO) in randomized order. Almonds were non-pareil, dry-roasted and added to the farina whole [provided by the Almond Board of California (Modesto, CA)]. Almonds and their processed forms were from the same almond harvest. Test breakfasts were matched on available carbohydrate and sweetness (nutrient composition shown in Table 2). The amount of almonds added to the cereal was 42.5 grams (~33 almonds) in accord with the Food and Drug Administration (FDA) qualified health claim regarding daily nut intake [13]. After completion of the breakfast meal, palatability of the foods was rated on a VAS (mean palatability scores are shown in Table 3).

**Table 2:** Test breakfast and lunch nutrient composition

	Vehicle	Whole	Almond	Almond	Almond	Lunch
		Almond	Butter	Flour	Oil	
Energy (kcal)	347	580	580	377	537	374

Weight (g)	644.0	683.5	674.9	656.9	665.5	393.6

Fat (g)	1	22.6	22.6	1	22.6	1.6

Protein (g)	7	16	16	16	7	11.4

Dietary fiber (g)	2.1	7.1	7.1	3.6	2.1	2.8

Soluble fiber (g)	1.4	1.9	1.9	1.5	1.4	1.3

Insoluble fiber (g)	0.7	5.2	5.2	2.1	0.7	1.5

Available carbohydrate (g)	75	75	75	75	75	75

**Table 3:** Mean palatability scores for test foods¹

	Vehicle	Whole	Almond	Almond	Almond
		Almond	Butter	Flour	Oil
Cereal	0.56 ± 0.06^a^	0.61 ± 0.07^a^	0.57 ± 0.07^a^	0.52 ± 0.08^a, b^	0.37 ± 0.08^b^

Orange Juice	0.75 ± 0.05	0.78 ± 0.05	0.73 ± 0.06	0.77 ± 0.04	0.73 ± 0.05

Bagel	0.59 ± 0.06	0.64 ± 0.06	0.58 ± 0.04	0.66 ± 0.05	0.65 ± 0.05

¹ Different superscripts indicate significant difference within the meal period (P < 0.05); values represent the score on a 100 mm visual analog scale.

Blood was drawn and appetite was rated 15, 45, 60, 90, 120, 180, and 240 minutes after completion of the test breakfast. At minute 240, participants consumed a standard lunch within 10 minutes that consisted of a plain white bagel, grape or strawberry jelly, and tap water (250 ml). Palatability of the lunch was rated on a VAS. Afternoon blood sampling and appetite scoring occurred using the same time intervals as the morning.

Three milliliters of blood were collected in a red top vacutainer at each draw. After clotting and centrifugation, serum was aliquoted and stored at -80°C for later analysis of insulin, glucose, and NEFA. Four ml of blood were collected in ice-cooled EDTA-plasma vacutainer, 40 μl DPP-IV inhibiter (Millipore, St. Charles, MO) was added, and samples were handled according to manufacturer's directions for analysis of GLP-1. All samples were analyzed in duplicate. Insulin and glucose were measured by electrochemiluminescence and the hexokinase method, respectively. Sensitivity of the insulin immunoassay was 1.39 pmol/l (within-run coefficient of variation (CV) of 1.9%). Hexokinase sensitivity was 0.12 mmol/l (within-run CV of 0.41%). NEFA were analyzed with an enzymatic colorimetric method on an automated analyzer with a sensitivity of 0.00014 mEq/L (within-run CV of 0.75%). GLP-1 was assessed by radioimmunoassay. Sensitivity of the assay was 3 pmol/l (within-run CV of 30.3%). Lipid panel assessment was conducted by MidAmerica Clinical Laboratories, Indianapolis, IN.

Nutrient data were analyzed with the Nutrition Data Systems for Research 2008 (University of Minnesota, Minneapolis, MN). Statistical testing was conducted with SPSS, Version 17.0 (SPSS Inc., Chicago, IL). Repeated measures analysis of variance (ANOVA) was used to explore main effects and, when appropriate, *post hoc *analyses were conducted with Bonferroni adjustment. Significance was set at p < 0.05. Data are represented as Mean ± SEM. Area under the Curve (AUC) with respect to increase (I) was computed to measure concentration change over time [14] (Formula 1) and quantitative insulin sensitivity check index (QUICKI) was calculated [15].

Fasting concentrations served as baseline for daylong (0-490 minutes) and morning responses (time 0-240 minutes) and the blood sample taken at 240 minutes served as the baseline for afternoon responses (time 240-490 minutes).

Formula 1. Incremental Area Under the Curve

$$AUCI=\left(\sum_{i=1}^{n-1}\frac{(m_{i}+m_{\left(i+1\right)})\cdot t_{1}}{2}\right)-\left(m_{1}\cdot \sum_{i=1}^{n-1}t_{i}\right)$$

Where n equals the total amount of measurements, m_i _equals the individual measurements, and t_i _equals the time between measurements [14].

## Results

### Blood glucose

**Figure 1:**
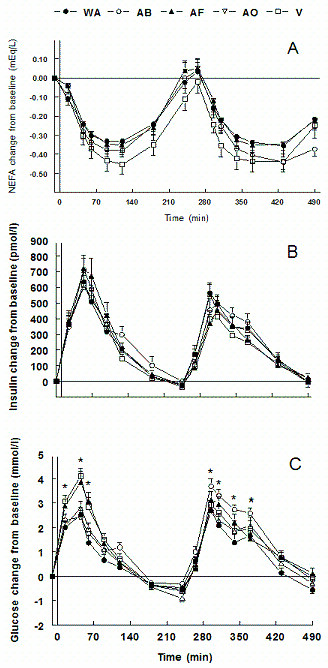
A) Daylong NEFA concentrations as change from baseline; B) Daylong insulin concentrations as change from baseline; C) Daylong glucose concentrations as change from baseline (*P < 0.05). black circles = WA (whole almonds); white circles = AB (almond butter); black triangles = AF (almond flour); white triangles = AO (almond oil); white squares = V (vehicle).

Fasting blood glucose concentrations were similar across treatment arms. Mean morning glucose concentrations were greater after consumption of V as compared to WA, AB, and AO (P < 0.02). Afternoon response was greater with AB than all others (P < 0.002). Mean daylong blood glucose change from baseline was lower after consumption of WA compared to AB, AF and V (P < 0.02) (Figure 1C). Daylong blood glucose response was lower with WA compared to V (P < 0.05).

V resulted in a greater blood glucose peak than AB and AO (P < 0.001) with a trend in comparison with WA (P = 0.069). The post-lunch peak was greater with AB compared to WA, AF, and V (P < 0.02) and approached significance compared to AO (P = 0.055).

**Figure 2:**
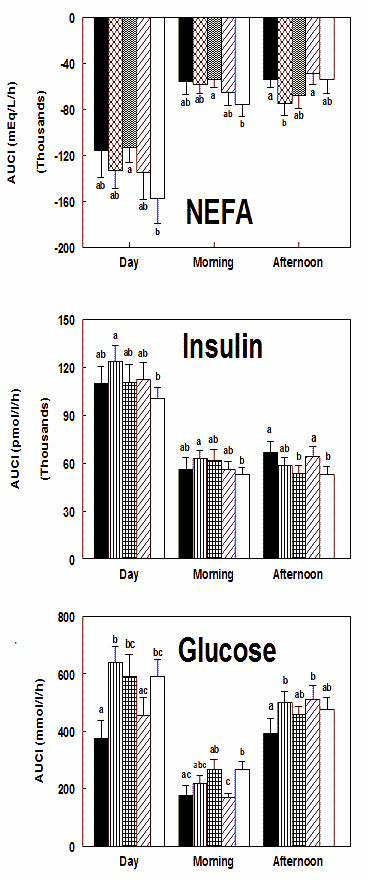
**A) NEFA AUCI for the day (time 0-490 min), morning period (first-meal; time 0-240 min.), and afternoon AUCI (second-meal; time 240-490 min.); B) Insulin AUCI ; C) Glucose AUCI. Different lowercase letters indicate significant differences within the response period for each measurement (P < 0.05).** (solid black = WA (whole almonds); vertical lines = AB (almond butter); vertical and horizontal lines = AF (almond flour); diagonal lines = AO (almond oil); solid white = V (vehicle)).

AUCI was less in WA compared to AB, AF, and V and greater in AB versus AO (P < 0.05) (Figure 2). Morning AUCI with AO was lower than AF and V (P < 0.01) and WA was lower than V (P < 0.04). Afternoon AUCI was lower after WA compared to AB and AO (P < 0.04).

### Serum Insulin and Insulin Sensitivity

Fasting insulin was greater in WA compared to V and AF; AO was greater than AF and AB (P < 0.05). The change of insulin from baseline in the morning and over the test day did not differ between treatments. The afternoon insulin response to V was less than WA, AB, and AO, whereas both AO and WA were greater than AF (P < 0.04) (Figure 1B). No differences in absolute peak concentrations occurred in the morning, but the afternoon peak was greater with WA compared to AF and AO compared to AF and V (P < 0.02).

AUCI was greater in AB versus V (P < 0.03) (Figure 2). Postprandial breakfast AUCI for AB was greater than V (P < 0.04). WA postprandial lunch AUCI was greater than AF and V (P < 0.05), and AF was less than AO (P < 0.01).

WA QUICKI was less than that for AB and AF (P < 0.05) and trended lower compared to V (P = 0.068). AO QUICKI was less than AB and AF (P < 0.02).

### Nefa

Fasting NEFA concentrations with AF were lower than AB and V (P < 0.02) and approached significance compared to AO (P = 0.06). Morning NEFA concentrations were greater with AB than all others (P < 0.05). AB was greater than V in the afternoon (P < 0.01). Daylong concentrations were lower with AF compared to both AB and AO (P < 0.03) and V was less than AB (P < 0.001) (Figure 1A). Absolute peak response in both time periods was greater in AB than in AF (P < 0.05). WA morning peak concentration tended to be lower than AB (P = 0.053).

Morning and daylong NEFA AUCI were less suppressed in AF versus V (P < 0.03) (Figure 2). NEFA AUCI following WA trended toward greater suppression than V in the morning and over the day (P = 0.067). Afternoon AUCI was less suppressed in AB versus WA and AO (P < 0.05).

### Plasma GLP-1

Fasting GLP-1 concentrations were similar between visits (19.99 ± 1.28 pmol/l). There were no significant treatment effects.

### Appetite Ratings

**Figure 3:**
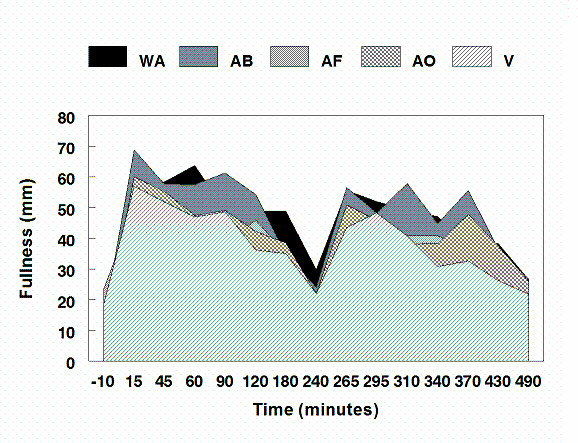
**Area plot of fullness ratings in millimeters from the "not at all anchor" of a 100 mm visual analog scale.** (WA = whole almond; AB = almond butter; AF = almond flour; AO = almond oil; V = vehicle).

No differences in fasting or treatment-related hunger ratings occurred. Daylong fullness ratings were significantly higher in WA compared to AF, AO, and V (P < 0.04) and in AB compared to AF and V (P < 0.01) (Figure 3). Fullness was greater in the morning with WA versus AF and AO and in AB versus AF (P < 0.04), with WA trending higher than V (P = 0.058). Afternoon fullness ratings were greater in WA compared to AF and V (P < 0.001), lower in AF versus AB (P < 0.01) and lower in V compared to AB and AO (P < 0.03).

### Palatability

Mean palatability was lower for the farina containing AO compared to WA, AB, and V (P < 0.04), but no sample rated in the lower third of the hedonic scale (Table 3).

### Body Weight and Composition

There was no difference in body weight, BMI, or any measures of body composition across treatment visits.

## Discussion

The aims of this study included confirmation that nut consumption improves the metabolic profile with respect to diabetes risk; determination of the relative contributions of different almond fractions on these indices and whether acute post-prandial benefits translate to improved insulin sensitivity at a subsequent eating event (second-meal effect). To enhance the ecological validity of the work, whole almonds were included to explore effects with natural mastication and the quantity of almonds included in the test meal corresponded to the recommended intake level in the FDA approved qualified health claim for nuts [13].

In the current study, significantly greater fasting insulin concentrations with WA and AO led to lower QUICKI values and therefore less calculated insulin sensitivity. Due to its correlation with the hyperinsulinemic euglycemic clamp [15], QUICKI is the preferred method for quantifying insulin sensitivity in populations with perturbed insulin sensitivity. Despite higher baseline insulin concentrations, consumption of WA and AO decreased morning blood glucose AUCI compared to V. Postprandial breakfast insulin and NEFA AUCI were not greater after consumption of WA and AO suggestive of greater insulin sensitivity (e.g. the decreased blood glucose AUCI was not determined by a concurrent increased insulin response). Similarly, consumption of 60 g of almonds with white bread decreased 2-hour blood glucose and insulin AUCI in healthy individuals compared to a control meal [7]. Moreover, in healthy men, bioaccessible almond composition produced a lower 3-hour blood glucose response with no significant difference in the insulin or NEFA response [9]. Larger almond particles did not produce the same effect. Previous data from our laboratory did not find a clear relationship between amount of almond chewing (predefined number of chews) and changes in glucose and insulin concentrations in a group of healthy participants [16]. Discrepancies between studies may be due to differences in almond particle sizes (e.g., naturally masticated versus predefined) which could alter nutrient bioaccessibility.

In contrast to lipid-containing treatments, the treatments with little fat (V and AF) produced the largest immediate postprandial glucose responses. The role of fat in decelerating gastric emptying may be partly responsible [17]. Although the AF treatment contained polyphenolic compounds, there was no evidence of impairment of starch digestion in the current study as has been previously reported [18]. NEFA concentrations after consumption of AF were lower than V in the morning postprandial period without differences in insulin concentrations, indicating a slight improvement in NEFA suppression. In comparison, no difference in NEFA concentrations between the combination of AF and AO, large almond particles, and control sunflower oil [9] suggests minimal benefit to the presence of the defatted flour fraction on metabolic risk outcomes.

One study suggested the NEFA concentration 4 hours after a test breakfast accounted for ~50% of the variability in the glycemic response to a standard lunch [19]. We found no significant difference at this time point and do not confirm that NEFA concentrations explain second-meal metabolic differences. However, AB resulted in the lowest overall degree of NEFA suppression in the morning period and was associated with the greatest blood glucose response to the standard lunch. The overall NEFA response in the period before the meal may be a stronger determinant of the second-meal glycemic response than the concentration immediately preceding the second meal. Additionally, no differences were observed in glucose, insulin, or GLP-1 concentrations at 4 hours after the test breakfast, suggesting other determinants of second-meal effects. While the mechanism remains uncertain, this trial confirms the phenomenon. Prior work revealed that inclusion of slowly digestible carbohydrate (e.g., lentils) in a breakfast meal reduced the glucose response after lunch [20]. We show that inclusion of a high unsaturated fat load with breakfast is also effective. Together, these data support the efficacy of dietary moderation of carbohydrate absorption kinetics from a morning meal for extended glycemic control in populations at risk for or with type 2 diabetes.

The high unsaturated fatty acid composition of almonds may account for the blunted glucose concentrations noted in the postprandial period. Acute consumption of PUFA and MUFA decreases postprandial glucose AUCI without altering insulin concentrations [21] due to increased efficiency of insulin receptor signaling and increased glycemic control through stimulation of GLP-1 [6]. Although no significant treatment effects were detected in GLP-1 concentrations, WA and AO led to an overall greater and sustained GLP-1 response that may have contributed to blunted second-meal blood glucose concentrations [22] and modified satiety [23].

The role of almond lipid bioavailability in triggering the release of gut peptides and contributing to energy balance is complicated by differences in the metabolic profiles following WA and AO versus AB consumption. Previous research in healthy participants showed lower breakfast and increased afternoon blood glucose AUCI after consumption of a standard lunch when peanut butter or butter was consumed in a mixed breakfast meal [24]. Our data show this similar afternoon rebound with consumption of AB in the breakfast meal, which cannot be attributed

solely to the lipid component. Additionally, *in vitro *gastric and duodenal digestion modeling found greater duodenal lipid digestion in finely ground almond particles compared to defatted finely ground almonds with almond oil added back, suggesting that differential dispersion of the lipid (e.g. different surface areas of the lipid droplets) may determine bioaccessibility [25]. Altering the physical form of nuts may have unexpected metabolic effects that warrant further investigation.

Differences in fullness were not likely due to variations in the macronutrient content of the test foods. All provided 75 g of available carbohydrate and the treatments matched on protein, fat, total and soluble fiber, and energy led to variable satiety responses. The cognitive influence of the visual cue of WA and the increased orosensory stimulation from chewing may be responsible for satiety effects [26]. Lipid consumed in oil form previously induced a greater and sustained CCK response and greater satiety in women versus consumption of WA [10], a finding not confirmed in the current study.

An unavoidable limitation of the current study was that breakfast meals were not matched on energy, volume, or macronutrient composition. Due to the study design, available carbohydrate (the main determinant of GI) was standardized between all treatments and macronutrients were matched when possible. The subjective palatability of the treatments was not consistent, with AO considered significantly less desirable than all other treatments except for AF. However, *post hoc *covariate analysis did not reveal palatability significantly altered results. Additionally, participants were instructed to consume the same meal before reporting for each visit, although significant differences were found. Fewer calories were consumed the night before WA compared to V (~200 kcal) and a lower percent of calories from carbohydrate was consumed the night before AO compared to AB (~5%). The extent to which these differences can explain postprandial breakfast responses is unknown. Greatly altering the GI of a dinner may produce a varied response after a breakfast meal, but the absorptive characteristics between the evening meals consumed before test days in the current study were unlikely so drastically different as those that have previously been shown to produce carry-over effects to the breakfast meal [27]. The possibility exists that differences in dietary intake may also be an artifact of the difficulty of accurately assessing dietary intake and single meal reporting precludes the ability to employ calculations such as the Goldberg cut-off to determine plausibility of reported intake. Nonetheless, the macronutrient and energy intake data appear to fall within normal ranges (49-54% energy from carbohydrate, 28-33% energy from fat, 18-19% energy from protein, and ~30% of mean estimated daily energy requirements).

In summary, inclusion of almonds in the breakfast meal of IGT adults decreased blood glucose concentrations and increased satiety acutely and after a second meal. The lipid component of the almond appears to be largely responsible for the immediate post-ingestive response, although it cannot account for the second-meal response. Overall, daylong glucose, insulin and NEFA concentrations were attenuated in the WA and AO treatments, indicating an improved hormonal profile with their consumption. Importantly, the absolute magnitude of the blood glucose-lowering response equals that achieved with acute administration of acarbose in individuals with IGT [28], suggesting the physiological relevance and applicability of the current findings.

## Competing interests

The authors declare that they have no competing interests. The funding body did not participate in the study design, data collection, analysis and interpretation of data, writing of the manuscript, or in the decision to submit findings for publication.

## Authors' contributions

AM and RM participated in the conception of the study. AM, RM, and RC participated in study design and data interpretation. AM participated in conduction of the experiment. All authors read and approved the final manuscript.
